# Physico-Chemical Properties of CdTe/Glutathione Quantum Dots Obtained by Microwave Irradiation for Use in Monoclonal Antibody and Biomarker Testing

**DOI:** 10.3390/nano14080684

**Published:** 2024-04-16

**Authors:** M. A. Ruiz-Robles, Francisco J. Solís-Pomar, Gabriela Travieso Aguilar, Maykel Márquez Mijares, Raine Garrido Arteaga, Olivia Martínez Armenteros, C. D. Gutiérrez-Lazos, Eduardo G. Pérez-Tijerina, Abel Fundora Cruz

**Affiliations:** 1 Centro de Investigación en Ciencias Físico Matemáticas, Facultad de Ciencias Físico Matemáticas, Universidad Autónoma de Nuevo León, Av. Universidad s/n, San Nicolás de Los Garza 66455, Nuevo León, Mexico; mitchel.ruizrb@uanl.edu.mx (M.A.R.-R.); claudio.gutierrezl@uanl.edu.mx (C.D.G.-L.); eduardo.pereztj@uanl.edu.mx (E.G.P.-T.); 2 Instituto de Ciencia y Tecnología de Materiales (IMRE), Universidad de La Habana, La Habana 10400, Cuba; gabriela.travieso@imre.uh.cu; 3 Instituto Superior de Ciencias y Tecnologías Aplicadas (InSTEC), Universidad de La Habana, La Habana 10400, Cuba; mmarquez@instec.cu (M.M.M.); abel.fundora@instec.cu (A.F.C.); 4 Grupo de Análisis, Instituto Finlay de Vacunas, Avenida 21 No. 19810, Atabey, Playa, La Habana 10400, Cuba; rgarrido@finlay.edu.cu (R.G.A.); omartinez@finlay.edu.cu (O.M.A.)

**Keywords:** quantum dots, murine myeloid cells, glutathione, cadmium telluride, microwave

## Abstract

In this report, we present the results on the physicochemical characterization of cadmium telluride quantum dots (QDs) stabilized with glutathione and prepared by optimizing the synthesis conditions. An excellent control of emissions and the composition of the nanocrystal surface for its potential application in monoclonal antibody and biomarker testing was achieved. Two samples (QDYellow, QDOrange, corresponding to their emission colors) were analyzed by dynamic light scattering (DLS), and their hydrodynamic sizes were 6.7 nm and 19.4 nm, respectively. Optical characterization by UV-vis absorbance spectroscopy showed excitonic peaks at 517 nm and 554 nm. Photoluminescence spectroscopy indicated that the samples have a maximum intensity emission at 570 and 606 nm, respectively, within the visible range from yellow to orange. Infrared spectroscopy showed vibrational modes corresponding to the functional groups OH-C-H, C-N, C=C, C-O, C-OH, and COOH, which allows for the formation of functionalized QDs for the manufacture of biomarkers. In addition, the hydrodynamic radius, zeta potential, and approximate molecular weight were determined by dynamic light scattering (DLS), electrophoretic light scattering (ELS), and static light scattering (SLS) techniques. Size dispersion and the structure of nanoparticles was obtained by Transmission Electron Microscopy (TEM) and by X-ray diffraction. In the same way, we calculated the concentration of Cd^2+^ ions expressed in mg/L by using the Inductively Coupled Plasma Atomic Emission Spectrometry (ICP-OES). In addition to the characterization of the nanoparticles, the labeling of murine myeloid cells was carried out with both samples of quantum dots, where it was demonstrated that quantum dots can diffuse into these cells and connect mostly with the cell nucleus.

## 1. Introduction

Quantum dots’ (QDs) emission wavelengths span over UV and IR spectra ensuring high quantum yield, high photostability, and high molar extinction coefficients [1,2,3]. Among the most prolific applications they can have in nanotechnology is nanobiotechnology, responsible for creating and using this type of material in diagnosis, treatment and monitoring in biological systems, as well as in the release of drugs [4], reducing side effects in benign areas and increasing the efficiency of signaling or monitoring of a specific site of the organism [5]. Among the various types of QDs, those synthesized in aqueous solutions [6] with relatively small sizes (2–6 nm) are employed as biomarkers owing to their size properties, specifically luminescence [7,8], and their biocompatibility [9,10,11,12], enabling them to be ideal candidates for biotechnological and medical applications. These luminescent nanoparticles have been the subject of much research in recent years. In addition, the ease of synthesis of these nanoparticles from almost any inorganic precursor and in aqueous solution provides the biotechnology industry with the opportunity to take advantage of their luminescent properties for various purposes, including their use as cellular biomarkers [13,14,15].

Compared to conventional organic fluorophores, QDs, specifically glutathione-stabilized CdTe-QDs, exhibit advantageous properties, including tunable emissions, photostability and high brightness [16,17]. These unique properties make different types of quantum dots highly desirable for certain biological applications and provide new possibilities for biological imaging and protein conjugations to detect polysaccharides [18,19]. QDs widely used in biolabeling were initially synthesized in the organic phase using high-boiling solvents and stabilizants such as trioctylphosphine oxide (TOPO) or reducers such as trioctylphosphine (TOP) [20,21]. Generally, these nanocrystals are capped with hydrophobic ligands. Therefore, to obtain biofunctional QDs, it is necessary to realize a change-phase process to transfer them to an aqueous solution and make them soluble in water [22,23]. Weller et al. pioneered the aqueous-based preparation of thiol-capped QDs [24]. So far, water-based synthesis with thiols as protective ligands has been developed as an interesting alternative [20,25]. Compared to conventional organometallic approaches, wet solution preparation is cost-effective and convenient, but generally results in a low quantum yield (QY) of 1 to 10% [26]. Of all the QDs reported to date, CdTe exhibits comparable optical properties to its organometallic analogs when prepared in water [20,25,26]. In the present research, glutathione (GSH) was chosen as a ligand to synthesize high-quality CdTe-QDs in an aqueous solution [27,28].

Highly stabilized CdTe quantum dots can be obtained taking advantage of the structure of the glutathione molecule [29]. The first advantage is the fact that glutathione contains the thiol group (R-SH), which is widely demonstrated to exhibit a strong chemical affinity with transition metals, such as cadmium. Consequently, the synthesis employs a majority cadmium precursor with respect to telluride precursor to favor the formation of a nanocrystal surface rich in cadmium ions. Then, the valence levels will be passivated by the thiol group, supplying the stabilization [30,31,32]. This surface is a natural mono-thiol compound that exists in most organs at the mM level and has no cytotoxicity [33]. In addition, surface modifications of GSH-coated QDs with biomolecules are relatively easy, because GSH has two functional groups, two carboxyl and one primary amino group, which can be conjugated to a biological compound such as an antibody [34]. Precisely, this property defines the other two advantages that the glutathione structure offers. The second advantage that the glutathione molecule offers is that it contains the carboxyl group (COOH) at its ends, which provides solubility in an aqueous medium, and the third advantage refers to the amine group site (NH_2_), which can interact with cysteine receptors present on myeloid cells. On the other hand, the COOH group of glutathione can bind to scavenger-like receptors (SRs) present on the surface of myeloid cells. Therefore, once bound to the receptors, glutathione-stabilized quantum dots and the molecules to which they have been bound are internalized by myeloid cells through a process of endocytosis. The labeling process of myeloid cells with glutathione has been used for the development of vaccines and cancer therapies. The COOH and NH_2_ functional groups of glutathione play a fundamental role in the labeling of myeloid cells. These groups allow glutathione to bind to specific receptors on the surface of myeloid cells, internalize, and activate the immune response. Glutathione can also modulate the immune response, depending on the concentration and the type of myeloid cell [35].

On the other hand, the advantages of preparing quantum dots with synthesis assisted by microwave irradiation are widely known [36,37,38]. This favors a thermal treatment directly on the nanocrystals by using a relatively simple method that requires shorter heating times with controlled vapor pressure in nearly inert conditions. This has allowed the preparation of a wide variety of compounds, including CdTe with a wide variety of organic stabilizants [39,40]; however, in the literature, it is not possible to find references to the microwave-assisted synthesis of CdTe quantum dots stabilized with glutathione. In this work, we have analyzed the synthesis and optical, structural and chemical characterization of CdTe/GSH-QDs. The novelty and advancements accomplished in this work are the study of the physico-chemical properties of CdTe/GSH-QDs and the use of microwave irradiation in their synthesis. These nanocrystals are used in biology and medicine to achieve cellular and biomarker testing [12,41,42,43,44,45,46].

## 2. Experimental Procedure

### 2.1. Materials

All reagents used, listed here, were from Sigma-Aldrich and used as received without further purification: Cadmium chloride (CdCl_2_, anhydrous ≥ 99%), Trisodium citrate dihydrate, L-glutathione (GSH, ≥98%), sodium borohydride (NaBH_4_, ≥96%), sodium tellurite (Na_2_TeO_3_, −100 mesh, 99%).

### 2.2. Synthesis of CdTe-GSH Quantum Dots

Luminescent CdTe/GSH-QDs were obtained from a precursor solution synthesized at room temperature following a facile one-pot method reported previously [47]. Briefly, a CdCl_2_ solution (0.04 M) was prepared in 40 mL of deionized water. This solution was added to 250 mL of deionized water under mechanical agitation. Then, 1000 mg of trisodium citrate dihydrate was added to the solution, maintaining the mechanical agitation; then, 500 mg of L-glutathione was added until a homogeneous solution was reached. Next, 10 mL of Na_2_TeO_3_ solution (0.01 M) was added to the mixture, and finally, 500 mg of NaBH_4_ was added until its complete dissolution.

To activate the fluorescence of CdTe/GSH-QDs, a post-synthesis treatment was realized by a microwave irradiation-assisted hydrothermal method, employing an Anton Paar Monowave 300 microwave reactor. Here, 20 mL of QDs solution was placed in a 30 mL G30 reactor followed by rapid heating with Monowave-300 up to 90 °C (for QDYellow) and 110 °C (QDOrange) and was maintained for 10 min. Thereafter, the reactor was rapidly cooled down to 50 °C.

The quantum dots were stored at 4 °C where the agglomeration of the nanocrystals was avoided, as well as possible cases of suspension, since the autoxidation of the thiol group was reduced, or where enzymatic reduction occurred of disulfides. This meant that the R-HS thiol group could derive into a disulfide, decreasing the stability of the QDs [48].

Although reports with glutathione establish a pH of 8 as optimal for the synthesis of CdTe-QDs, a general rule for establishing chemical bridges with other organic materials is not reported. Through studies prior to characterization, it was possible to determine that for the quantum dots analyzed, the optimal pH to develop a subsequent conjugation process should be in a range between 5 and 6. In this way, a high surface charge can be guaranteed and, with it, there is a greater possibility of anchoring to biomolecules. Due to this, and to guarantee that the QDs were bioconjugable, a surface charge of the nanoparticle greater than −20 mV was achieved, reducing the pH from 8 to 5. In this way, it is guaranteed that there is a greater number of functional groups on the surface with a negative charge that can bind to other functional groups that proteins have [49,50].

In this case, quantum dots show a molecular weight–particle size relationship, since a lower weight implies better purification. This is explained by the fact that the smaller this value is with respect to proteins, the greater the probability that the peaks of effectiveness of the conjugations will be narrower and more defined. Furthermore, a smaller size implies a lower number of ions and, therefore, a lower molecular weight of the nanocrystal [51].

### 2.3. Optical Absorbance Spectroscopy

For the optical absorbance measurements of QDs, aliquots of samples were added in a 10 mm quartz cuvette (Hellma Analytics, Müllheim. Germany) and placed in an Ultrospec 9000PC UV–Visible Spectrophotometer (Biochrom, Cambridge, UK). The Resolution CFR (Biochrom, version 3.2.0) software was used to collect and analyze the data.

### 2.4. Dynamic, Static and Electrophoretic Light Scattering

Dynamic Light Scattering (DLS), Electrophoretic Light Scattering (ELS) and Static Light Scattering (SLS) measurements were taken with an Anton Paar Litesizer^TM^ (Anton Paar ShapeTec GmbH, Wundschuh, Austria) 500 equipped with a 658 nm He–Ne laser operating at an angle of 90°. Scattering light detected at 90° was automatically adjusted by laser attenuation filters. The particle size distribution and molecular weight were measured in a 3 mm low-volume quartz cuvette (Hellma Analytics). The determination of zeta potential of the samples was carried out using an omega cuvette (Anton Paar). For data analysis, the viscosity, refractive index (RI) and relative permittivity of water (at 25 °C) were used. The Kalliope Professional (Anton Paar, version 2.18) software was used to collect and analyze the data. Each sample was characterized by DLS and ELS techniques using a concentration of 0.5 mg/mL. For the SLS measurements, each sample was prepared with a concentration of 3–9 mg/mL.

### 2.5. Transmission Electron Microscopy (TEM) and X-ray Diffraction (XRD)

CdTe/GSH-QDs were analyzed to determine their morphology, size dispersion and structure using a TEM JEOL-JEM-2010, with an acceleration voltage of 120 kV. Initially, the sample was cleaned by 5 cycles of centrifugation at 3000 rpm for 5 min in a 1:1 solution of ethanol and deionized water, and then 30 μL/mL aqueous solution was put on a mesh #200 TEM cupper grid.

X-ray diffraction (XRD) pattern was obtained on a PNalytical X’Pert^3^ Powder diffractometer with Cu κ_α_ radiation (λ = 1.5405 Å).

### 2.6. Photoluminescence Spectroscopy

The photoluminescence spectra were measured with a Hellma spectro fluorimeter FP-8200 (Hellma Analytics), where Hellma quartz cuvettes of the type 105.202-QS were used. The measurement was performed under an excitation wavelength of 466 nm. The detector was set to low sensitivity, and it was unnecessary to dilute the sample since the obtained spectra fit the desired intensity range.

### 2.7. FT-IR Spectroscopy

Surface composition analysis of samples was performed by Shimadzu IR Prestige 21 (Shimadzu, Tokyo, Japan). Aliquots of 5 mL of samples were deposited in a porcelain capsule and placed in an oven at room temperature for five days until the water content evaporated completely. Once the product was dried, 100 mg of crushed potassium bromide was taken and spread throughout the capsule to drag the microparticles of the solid and thus form the tablet that would be placed in the IR equipment. The FT-IR spectra of the sample were acquired in the wavelength range of 4000–600 cm^−1^.

### 2.8. Atomic Emission Spectroscopy (AES)

The AES analysis was realized in an Ultima Expert ICP-OES spectrophotometer with induction-coupled plasma, coupled to a polychromator with argon gas. Its optical system was thermally stabilized, with a 1 m focal length lens, 2400 g/mm and grating used in the 1st and 2nd order with an optical resolution less than 6 pm for 120–450 nm and less than 11 pm for 450–800 nm.

The spectral line used for Cd was 226.5 nm, which is free from interference. The water method (2023), which contains 25 elements placed in 50 mL volumetric containers with initial concentrations of 1000 mg/L, was used.

## 3. Results and Discussion

### 3.1. Particle Size

DLS is an optical method for the characterization of suspensions and emulsions. Analysis of the fluctuations of the scattered light gives information about the suspended particles. Fluctuations in the intensity of the scattered radiation are characterized by calculating the intensity compensation function, which ultimately allows for the evaluation of the diffusion coefficients of the particles. The quality of the result essentially depends on the quality of the data and the parameter settings. Modern dynamic light scattering devices automatically perform an intensity analysis of the compensation function and calculate the diffusion coefficient [33].

The diffusion coefficient *D* is related to the radius of the nanoparticles using Equation (1):

(1) $$R_{h}=\frac{k_{B}T}{6\pi \eta D}$$

where $R_{h}$ is the hydrodynamic radius, $k_{B}$ is the Boltzmann constant, *T* is the absolute temperature, *η* is the mean viscosity, and D is the translational diffusion coefficient [52].

The hydrodynamic radius of the samples prepared via both methods is given in Table 1. The radius of QDYellow was 6.70 nm and it was 19.4 nm for QDOrange.

**Figure 1:**
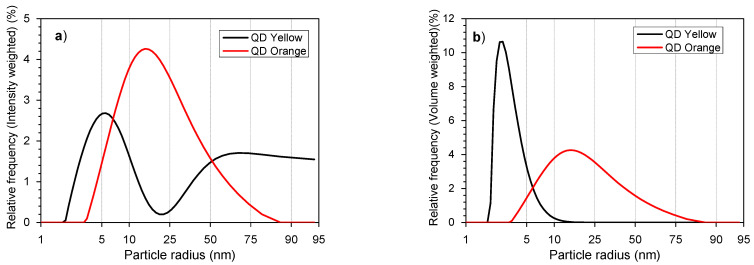
Average size distribution of QD samples. (**a**) Intensity distribution, (**b**) volume distribution.

Figure 1a,b show distributions of the hydrodynamic radius value for the analyzed samples with the intensity and the volume graphs. We can see that one of the size populations seen on the intensity graph disappeared in the volume graph. We consider that the particles are dispersed and have minimal agglomeration in the solution. This measurement includes the hydrodynamic size of the compound in which the nanoparticle itself (nucleus/shell) is included and a part of the ionic atmosphere that moves with it [53]. Likewise, the notable difference in nanoparticle size between the QDYellow and QDOrange samples could be due to the binding of glutathione molecules with the shell of the same stabilizer deposited on the surface of the quantum dot.

During the measurements, nanocrystals can absorb some of the transmitted frequencies, mainly the larger ones, which do not exhibit luminescence. But due to this absorption, it is possible to record variations in the intensity of the scattered laser light, which is why the large size is predominant for the QDOrange sample. It should be noted that the recorded size not only includes the solid phase of the nanocrystal but also absorption sites of the organic shell and the ionic environment of the nanocrystal itself.

### 3.2. Transmission Electron Microscopy and X-ray Diffraction

**Figure 2:**
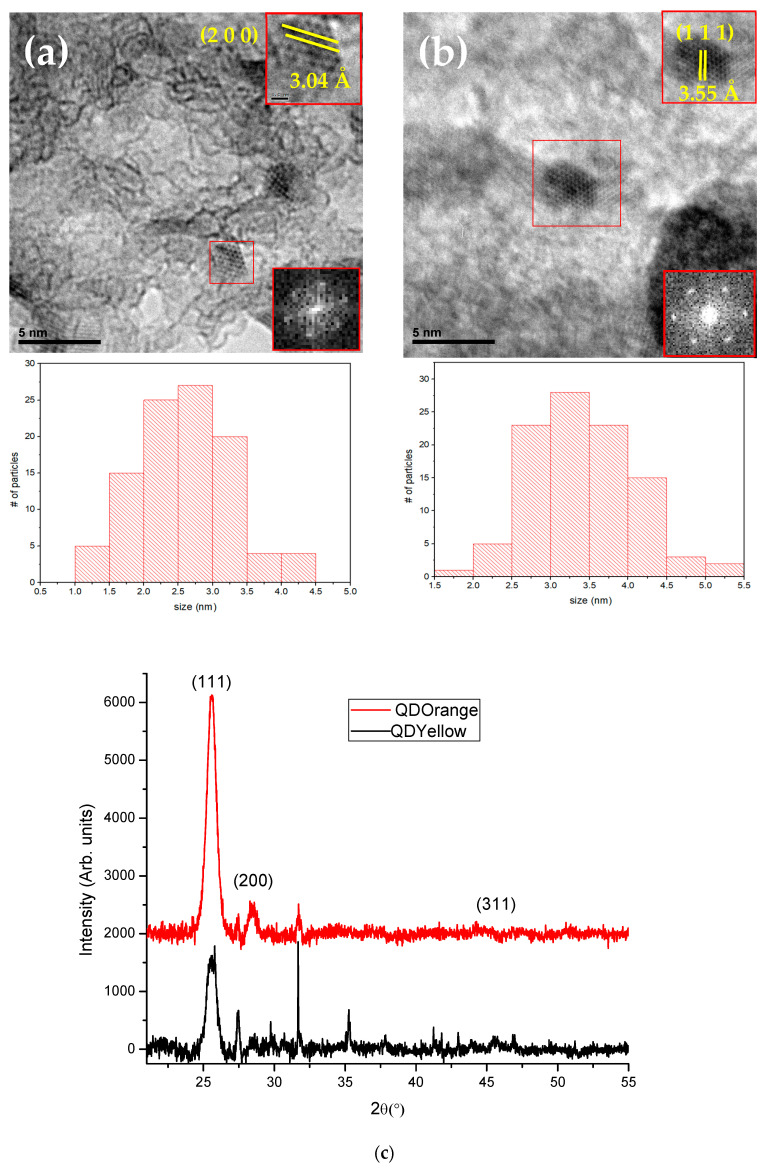
HRTEM images and size distribution of (**a**) QDYellow sample, (**b**) QDOrange sample, (**c**) XRD patterns of both samples (ICSD-093942).

The morphology and structural properties of the CdTe/GSH-QD samples were inspected by TEM at 800,000×. Despite the fact that the micrographs exhibited a slight agglomeration of nanocrystals, a homogeneous mean size distribution was obtained after measuring 100 nanoparticles for each sample with a mean size of 2.5 nm for the QDYellow sample and 3.4 nm for the QDOrange sample. The crystallinity of the CdTe/GSH-QDs was indexed with the same fcc crystal phase of CdTe for both samples as previously reported [43]. The insets of the TEM images show a spacing of 4 Å of the (200) plane family for the QDYellow sample (Figure 2a) and a 3.55 Å spacing of the (111) plane family for the QDOrange sample (Figure 2b). Figure 2c contains the XRD patterns of both samples. The two down-right insets show the fast Fourier transform (FFT) of the selected zone in red, and indicate the excellent crystallinity of both samples. In Figure 2c, the XRD patterns of QDYellow and QDOrange are shown. The nanocrystals exhibited diffraction peaks at 25.5°, 28.5° and 45.7°, which are slightly shifted with respect to the position of the diffraction peaks belonging to the most stable crystalline phase of CdTe [44]. Various reports indicate that this shift is due to induced stresses by the GSH stabilization, possibly due to the incorporation of sulfide ions into the CdTe nanocrystals [24,54,55].

### 3.3. Molecular Weight

Static Light Scattering allows us to determine the molecular mass and the second coefficient of the virial of solid phases dispersed in solution, such as colloidal systems. In this technique, the intensity of the scattered light is directly related to the molecular weight.

In a Litesizer system, the scattering intensity is measured in solutions with different concentrations and a Debye diagram is generated. The intercept of which at zero concentration provides the molecular weight (Zimm equation of the Rayleigh–Debye–Gans Model (Equation (2))) [56]. On the other hand, the second coefficient of the virial is a thermodynamic parameter that describes the interaction of the dissolved molecules with each other and with the molecules of the solvent. It is possible to determine this parameter from the Debye Diagram with Equation (2). Here, the second coefficient of the virial is the slope of the graph.

(2) $$\frac{KC}{R\left(\theta \right)}=\frac{1}{M_{W}P\left(\theta \right)}+2A_{2}C$$

where $M_{W}$ is the mean molecular weight, $A_{2}$ is the second virial coefficient, P(θ) is a corrective shape factor for large particles/molecules with respect to the laser wavelength, R(θ) is the Rayleigh relation, C is the concentration of particles and K is an optical constant.

The results obtained, shown in Table 2, indicate a first positive virial coefficient for QDYellow, which shows that the molecules of the QD tend to have a higher affinity for the molecules of solvents. That is, they are preferably surrounded by molecules of solvents and not by other segments of QDs. Whereas in the case of QDOrange, a negative coefficient was obtained, ascribing a greater affinity for other segments of quantum dots than between them and the molecules of solvents. This causes a structural rearrangement of the QD, becoming more compact and draining the solvent molecules. The difference in molecular weight values is owed to the presence of agglomeration in the sample and their own synthesis conditions. This difference in the molecular weight of the two samples is consistent with the results previously analyzed by light scattering, since the size of the nanocrystal is practically 3 times larger for QDOrange with respect to QDYellow, which means that the QDOrange sample contains a greater number of ions and therefore a higher molecular weight.

### 3.4. Fourier-Transform Infrared Spectroscopy (FT-IR)

**Figure 3:**
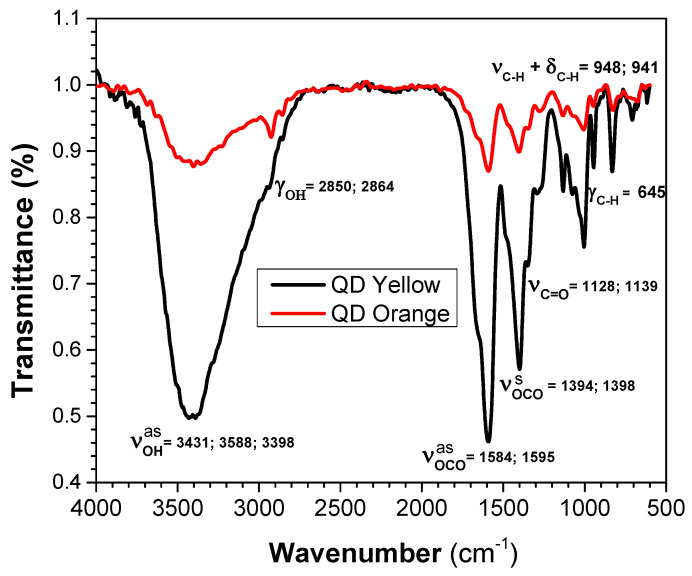
FT-IR spectra for CdTe/GSH-QDs.

The FT-IR spectra of the analyzed samples is given in Figure 3. The vibrational modes corresponding to functional groups of glutathione molecule are listed in Table 3. From the analysis, it is possible to determine the functional group that is coordinating the surface of QDs. It turned out to be the COOH group in equilibrium with the carboxylates, whose ionization makes the two C-O bonds equivalent [57]. On the other hand, when we used a stabilizer on the structure such as GSH, it was possible to determine that the functional group would be coordinating the core/shell of the internal structure of the QD [51].

With these results, it is verified that glutathione binds to the surface of the QD through the thiol group. When the thiol has been deprotonated, an R-S^−^ radical bonds strongly to the Cd^2+^ majority surface, although it still not accepted that a CdS shell is formed; this bond provides stability to CdTe-QDs against aggregation. In addition, the molar ratio between CdCl_2_ and Na_2_TeO_3_ is 4:1. It is expected that the surface of the QDs will have mostly Cd^2+^ ions, and the solubility of the nanocrystal will depend on the carboxyl groups. The amine bonds are terminals that allow the functionalization of the quantum dots with other anions (molecules, proteins, enzymes, etc.).

### 3.5. Zeta Potential

Electrophoretic Light Scattering (ELS) is a method used to measure electrophoretic mobility through Doppler shifts in scattered light. The electrophoretic mobility $\mu_{E}$ is defined in Equation (3)

(3) $$\mu_{E}=\frac{\nu }{E}$$

where ν is the particle electrophoretic velocity, and E is the applied electric field.

The zeta potential of particles in suspensions ζ is derived from the measured electrophoretic mobility, using Equation (4) (Henry equation with Smoluchowski approximation).

(4) $$\zeta =\frac{3\eta \mu_{E}}{2\varepsilon }$$

where ε is the relative permittivity and η is the dynamic viscosity [58].

**Figure 4:**
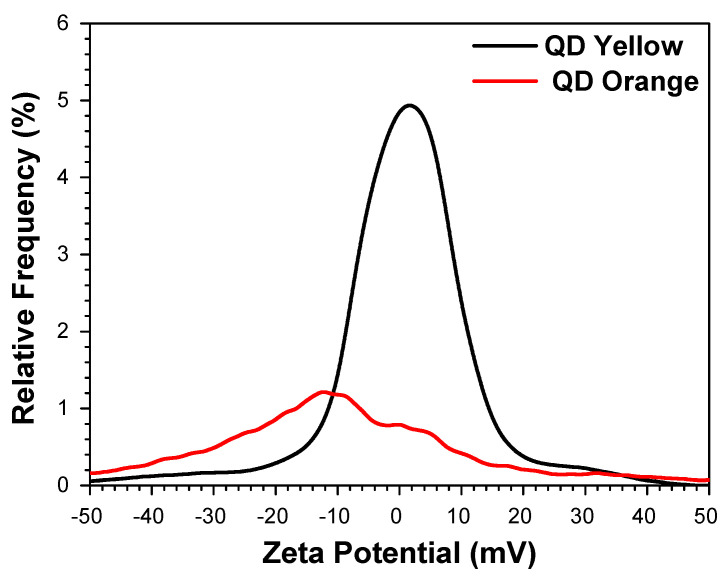
Determination of the average zeta potential.

The results of ζ potential measurements are presented in Figure 4. Infrared spectroscopy demonstrates that the carboxyl group is responsible for the negative charge of QDs in the solution due to it dissociating into carboxylate ions. Therefore, it remains outside the coordination sphere and free for conjugation. Simultaneously, inside the nanocrystals, the R-SH^−^ group is found coordinating with the S^2−^ ions generated possibly from the rupture of Cd-S. Consequently, zeta potential values were obtained for the QDYellow sample of −6.6 mV and −16.8 Mv for QDOrange. The charge concentration for both samples is due to the difference in charge existing between the surface, i.e., the solvation layer, and the interior of the nanocrystals [58]. The negative values indicate the excess of negative charges that exist on the surface of both samples, which can be associated with the presence of the conjugated form of the carboxyl: COOH ⇄ COO^−^.

The presence of negative charges associated with the strong coupling of oscillators between the resonant structures constitutes a crucial factor for future chemical to biomolecular bonds, such as the union with an NH_2_ group of proteins such as antibodies to form an amide bond.

### 3.6. Optical Absorbance Spectroscopy (UV-Vis)

**Figure 5:**
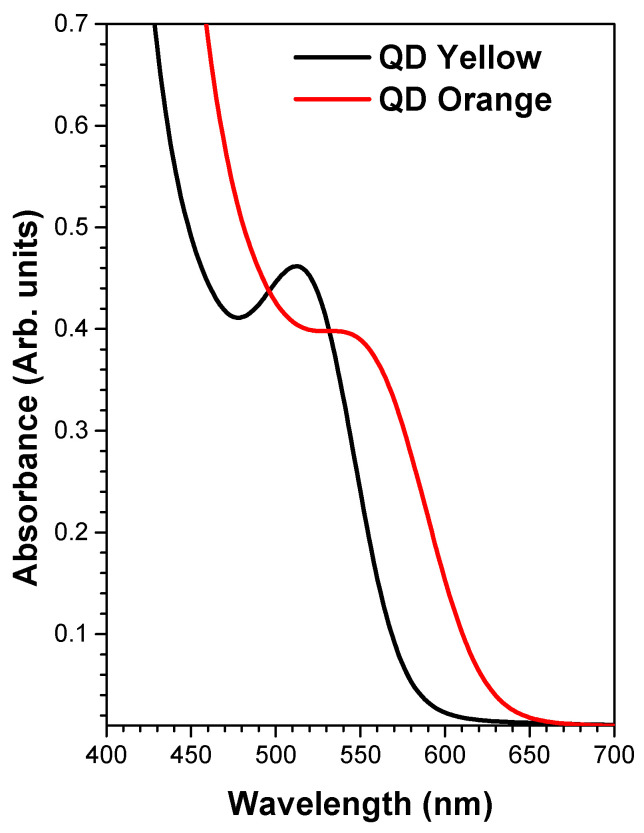
Absorbance spectrum of QDYellow (black line), of which the maximum excitonic peak is localized around 517 nm, and of QDOrange (red line), of which the maximum of the excitonic peak is localized around to 554 nm.

Figure 5 shows the absorbance spectra of both CdTe/GSH-QDs samples. The slight separation in the position of the excitonic peak is due to the difference in nanocrystal size, and the widening of the excitonic peak is related to their size dispersion [59].

The relationship between the maximum of the excitonic peak with the size of quantum dots [60] helps us to understand the coating extension of the glutathione shell on the QD surface. The very close position between the maxima of the excitonic peaks of the QDOrange sample and QDYellow suggests the formation of an extensive glutathione shell in the QDOrange sample, since the nanocrystal size measurement by DLS was 19.4 nm.

### 3.7. Photoluminescence

**Figure 6:**
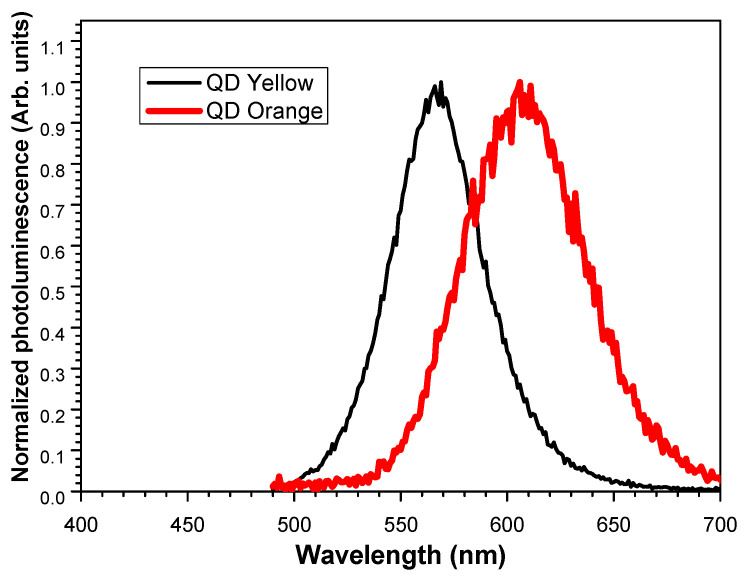
Normalized emission spectra corresponding to analyzed samples. For QDYellow, the maximum wavelength of emission was 570 nm, under an excitation wavelength of 472 nm. For QDOrange, the maximum wavelength of emission was 606 nm, under an excitation wavelength of 473 nm.

The ability of CdTe-QDs to be strongly luminescent lies in their ability to absorb UV radiation, which means that they are excellent absorbers in the UV spectral range [61]. The normalized photoluminescence spectra in Figure 6 unveil the emission properties of the QDs and corroborate the maximum emission wavelengths for each of the samples.

It exhibits a well-defined peak, within the interval of 570–590 nm (corresponding to the yellow color) and 590–620 nm (orange) [62]. The maximum intensity peaks are at 570 nm and 606 nm, respectively, which produces an emission very close to orange. The photoluminescence spectra are a characteristic measure of the quality of the synthesis and size distribution of the prepared nanocrystals [59].

For both samples, the maximum peak of luminescence is shifted to a higher wavelength with respect to the maximum peak of the excitation source, which was 473 nm. So, any contribution of the excitation source in the emission spectrum obtained can be neglected.

### 3.8. Inductively Coupled Plasma–Atomic Emission Spectrometry (ICP-OES)

To perform the ICP-OES measurements, four standard solutions of known concentrations were prepared in a volume of 100 mL, to which the cadmium standard substance was added in concentrations of 0.5; 1.5; 3.0; 6.0 mg/L, respectively. The working dilution was 1:100 by adding distilled water starting from 500 µL up to 50 mL for each QD. The final concentrations of Cd^2+^ obtained in both samples are summarized in Table 4.

For the synthesis of CdTe quantum dots, a nominal concentration of cadmium chloride [CdCl_2_] = 0.04 M, with a molecular weight of MW = 183.32 g/mol, was used. If we subtract the mass of the two chlorine atoms, we have MW = 112.414 g/mol of Cd^2+^ alone. This represents a nominal mass in the CdTe synthesis of

$$\left[{\mathrm{Cd}}^{2+}\right]=0.04\;\mathrm{mol}\;112.414\;g/\mathrm{molL}=4.49\;g/L$$

A nominal content of 4.49 mg/mL of cadmium was applied to the synthesis; for 40 mL of solution in deionized water, a nominal total of 179.6 mg was used.

**Figure 7:**
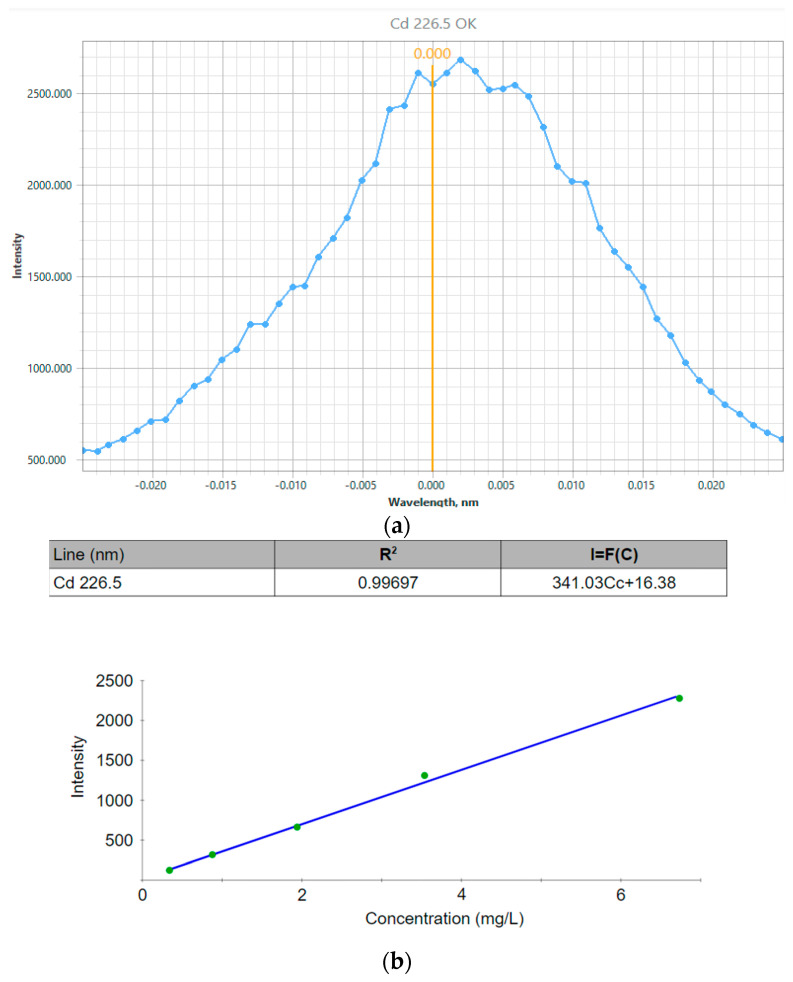
(**a**) Analytical signal for Cd^2+^, (**b**) calibration curve for Cd^2+^.

Given the considerable values obtained, the presence of Cd^2+^ in both samples is confirmed. Furthermore, the appearance of the analytical signal for Cd^2+^, as well as the calibration curve obtained, was suitable for the analysis, with a correlation coefficient higher than 0.99 as elaborated in Figure 7a,b. Thus, the presence of Cd^2+^ ions is verified by the high concentration values obtained.

Table 5 summarizes the main results obtained for the measurements taken.

According to the particle size obtained by the DLS and QELS techniques within a range of 6–20 nm, the values of the emission, excitation and absorption wavelengths correspond to quantum dots with emissions of yellow and orange, according to what is reported by the literature [59]. Furthermore, the low molecular weight determined by the SLS technique is proportional to the nominal concentrations of Cd^2+^ ions and those determined by atomic emission spectrometry. It is considerably feasible to establish possible conjugation strategies with different proteins such as monoclonal antibodies, since it would be possible to apply a method of purification and separation of excesses in a simple way, due to the considerable difference between the molecular weight of the antibodies (~180 kDa), which is higher than that of the QDs.

Additionally, the results of the valence vibrations and the characteristic bands obtained in the IR spectrum correspond to the negative values of zeta potential recorded through electrophoretic light scattering. This is strongly associated with the intense coupling of oscillators due to the COO- ⇆ COOH resonant structures coming from glutathione. Since there is a free carboxylate group in the coordination sphere of the nanoparticle, the possibilities of conjugations are not restricted, but rather the probability of forming chemical bonds with other biomolecules is considerably high.

**Figure 8:**
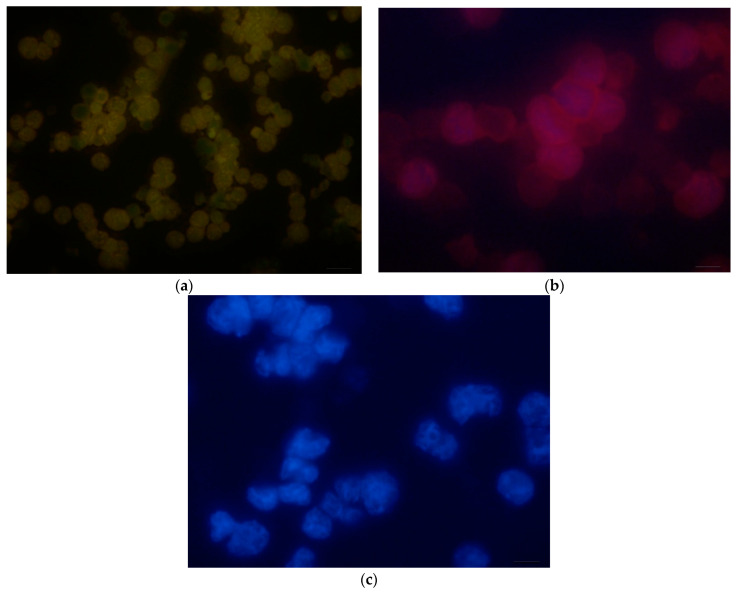
Micrographs of murine myeloid cells labeled with quantum dots. (**a**) Myeloid cells labeled with CdTe/GSH/Yellow with 40× green filter. (**b**) Myeloid cells labeled with CdTe/GSH/Orange with 40× red filter. (**c**) Myeloid cells labeled with DAPI 100×.

On the other hand, once the samples were characterized, the design, preparation and testing of the labeling of murine myeloid cells with quantum dots was carried out. To obtain the high-resolution images shown in Figure 8, a fluorescence optical microscope was used. The cells were placed on a glass slide fixed in cold acetone for 30 min. For their preparation, the electroporation internalization method was used fixed in acetone to permeabilize them with 30% of cold methanol for 30 min. Two washes were performed with 1X PBS before and after their permeabilization. The incubation time was 2 h at a temperature of 27 °C.

The quantum dots were added drop by drop into each of the circular regions that contain the cells. In Figure 8a,b, images taken of one of the fields of these cells marked with each type of quantum dot are shown. One of these regions, which was prepared only with 4′,6-diamidino-2-phenylindole or DAPI, was taken as a positive control (Figure 8c). This nuclear contrast dye is commonly used as a colored fluorescent nucleic acid blue stain which binds strongly to the regions rich in adenine and thymine in the minor groove of the double-stranded DNA molecule to localize the position and shape of the nuclei [63] and at the same time serves as a contrast with the respective emission colors of quantum dots.

Once the luminescent nanoparticles are in the cells, they will be in contact with multiple and widely varied biomolecules, including proteins, lipids, saccharides, etc., which will immediately adhere to the surface of these nanoparticles (forming a commonly corona-named structure), giving it a totally different biological identity. This causes the interaction with the cell membrane (CM) to be influenced by variations on the specificity of the nanoparticles, modifying the internalization mechanisms and/or activating different intercellular signaling pathways [64].

Quantum dots, once internalized, can take different routes within the cell. For example, when interacting with human macrophages, they are rapidly endocytosed, and subsequently they are directed by active cytoplasmic transport to the nucleus, which they enter through the nuclear pore complexes until they reach the nucleosome, where they accumulate [65].

In general, it was observed that both the nucleus and the cytoplasm of the cells were marked with quantum dots, since these act as a visual indicator for the hybridization of the complementary DNA sequences with the target of the quantum dot. Furthermore, DNA binding molecules are capable of targeting and binding nanomaterials to DNA in cell nuclei. The above could be explained because glutathione is the organic molecule that binds to DNA either by binding to the major and/or minor groove, intercalation, binding to the main phosphate chain and/or DNA alkylation. The different DNA binding agents each target the nanomaterial to DNA, but the affinity of the DNA binding agent determines the staining profile of DNA in the nucleus [66]. In the part that joins the cytoplasm, it stains irregularly due to the appearance of small RNA inclusions and the accumulation of waste substances. Furthermore, DNA is contained in a specific region of the cytoplasm, called the nucleoid, in most cases without being separated by the membrane [67].

Another fact that could explain this labeling is due to the nanometric size of QDs, allowing the nanocrystals to cross the cell membrane and the organelle membranes, where once internalized, their presence can be detected, as shown in Figure 8a,b, which shows similarity with the previously stated results, since these show great affinity with molecules such as DNA and RNA, joining through strong interactions. Their size directly affects the effectiveness of the internalization mechanism and its kinetics, without leaving aside their chemical nature. Their very small dimensions initially accumulate on the cell membrane and subsequently enter the cell gradually [65].

In general, quantum dots are encapsulated in vesicles and internalized either by active mechanisms or by passive penetration into the inner cell. However, internalization is not the only way in which these nanoparticles exert an effect inside the cell since their interaction with different membrane receptors can trigger very different and varied in-tricellular signaling pathways [68]. Furthermore, they contribute to defining the nucleus, delineating its boundary and establishing its morphology.

## 4. Conclusions

The physicochemical properties of glutathione-stabilized quantum dots analyzed in this research indicate that nanocrystals can be useful for the detection of tissues and malignant tumors smaller than 1 cm in size. The hydrodynamic radius of CdTe-QDs was reported with higher values than that reported by TEM microscopy, possibly due to the agglomeration of glutathione molecules on the nanocrystal surface. Additionally, glutathione tends to crystallize and then the diffraction patterns of the samples exhibit highly intense peaks due this organic phase, making it necessary to subtract this signal to appreciate the diffraction of the inorganic phase of CdTe. The Inductively Coupled Plasma–Atomic Emission Spectrometry results allow us to verify that glutathione-stabilized CdTe quantum dots not only internalize into the cells but also mark the cytoplasmic, nuclear and surrounding regions in the cytoplasmic membrane. This was shown through a strong interaction between the DNA and RNA present in the cells with the quantum dots, constituting an example of their effectiveness as cellular biomarkers, when observing their luminescence by fluorescence optical microscopy. According to the presented data from this report, we can conclude that CdTe-QDs are stabilized with glutathione, whose synthesis is assisted by microwave irradiation; they can be applied in both nuclear and cytoplasmic labeling, which could allow a more precise diagnosis of diseases such as leukemia, lymphoma and other myeloproliferative disorders. Additionally, they could be used to target specific drugs or therapeutic agents to myeloid cells, which would increase the effectiveness of the treatment and reduce side effects.

## Figures and Tables

**Table 1:** Hydrodynamic radius is determined by DLS. The size reported is derived from the volume graph.

	DLS	
	QDYellow	QDOrange
*R_h_* (nm)	6.70	19.37

**Table 2:** Results obtained by static light scattering.

Aspects	Debye Method	
	QDYellow	QDOrange
$M_{w}$ (Da)	314	969
$A_{2}$ (mol × mL/g^2^)	7.44 × 10^−1^	−8.44 × 10^−3^

**Table 3:** Assignment of the bands corresponding to GSH molecule shown in the IR.

Assignment	Wavenumber (cm^−1^)	
	QDYellow	QDOrange
$\nu_{OH}$	3431; 3388; 2935	3508; 3398; 2922
$\nu_{C-N}$	1280	1267
$\nu_{C-O}$	1128; 1074; 1001	1139; 1001
$\nu_{C-H}\;$+$\;\delta_{C-H}$	2850	2864
$\nu_{\mathrm{oco}}^{\mathrm{as}}$ $\nu_{\mathrm{oco}}^{s}$	1595; 1394	1584; 1398
$\gamma_{\mathrm{NH}}$	835	821
$\gamma_{\mathrm{OH}}$	948	941
$\gamma_{C-H}$	703	675

**Table 4:** Results of the analysis by ICP of the samples analyzed.

Sample	Size	Cd^2+^ (mg/L)
QDYellow	6.70 nm	5.246
QDOrange	19.37 nm	5.198

**Table 5:** Main results.

Aspects	QDYellow	QDOrange
Particle Size (DLS)	6.70 nm	19.4 nm
Particle Size (QELS)	9.30 nm	19.6 nm
Molecular Weight (Debye Method)	314 Da	969 Da
2nd Virial coefficient (Debye Method)	7.44 × 10^−1^ mol × mL/g^2^	−8.44 × 10^−3^ mol × mL/g^2^
Mean zeta Potential	−6.6 mV	−16.8 mV
Emission, excitation and absorption wavelength	λ_em_. = 570 nm λ_ex_. = 472 nm λ_abs_. = 517 nm	λ_em_. = 606 nm λ_ex_. = 473 nm λ_abs_. = 554 nm
Main functional groups	3431 cm^−1^ $\left(\nu_{OH}^{asoc}\right)$, 1595 cm^−1^ $\left(\nu_{oco}^{as}\right)$, 1394 cm^−1^ $\left(\nu_{oco}^{s}\right)$	3508 cm^−1^ $\left(\nu_{OH}^{asoc}\right)$, 1584 cm^−1^ $\left(\nu_{oco}^{as}\right)$, 1398 cm^−1^ $\left(\nu_{oco}^{s}\right)$
Cd^2+^ concentration	5.246 mg/L	5.198 mg/L

## Data Availability

All data are available as part of the article.
